# Rabies vaccine initiation and adherence among animal-bite patients in Haiti, 2015

**DOI:** 10.1371/journal.pntd.0006955

**Published:** 2018-11-13

**Authors:** Cuc H. Tran, Maxwell Kligerman, Lesly L. Andrecy, Melissa D. Etheart, Paul Adrien, Jesse D. Blanton, Max Millien, Ryan M. Wallace

**Affiliations:** 1 Epidemic Intelligence Service, Division of Scientific Education and Professional Development, U.S. Centers for Disease Control and Prevention, Atlanta, Georgia, United States of America; 2 Poxvirus and Rabies Branch, Division of High-Consequence Pathogens and Pathology, National Center for Emerging and Zoonotic Infectious Diseases, U.S. Centers for Disease Control and Prevention, Atlanta, Georgia, United States of America; 3 Stanford University, Department of Otolaryngology, Head and Neck Surgery, Palo Alto, California, United States of America; 4 Family Health Ministries, Durham, NC, United States of America; 5 Ministère de la Santé Publique et de la Population, Direction d’Epidemiologie de Laboratoire et de Recherche, Port-au-Prince, Haiti; 6 US Centers for Disease Control and Prevention, Port-au-Prince Prince, Haiti; 7 Ministère de l'Agriculture, des Ressources Naturelles et du Développement Rural, Port-au-Prince, Haiti; Wistar Institute, UNITED STATES

## Abstract

**Background:**

Approximately 59,000 people die from rabies worldwide annually. Haiti is one of the last remaining countries in the Western Hemisphere with endemic canine rabies. Canine-mediated rabies deaths are preventable with post-exposure prophylaxis (PEP): wound treatment, immunoglobulin, and vaccination. In countries where PEP is available, variability in healthcare seeking behaviors and lack of adherence to recommended treatment guidelines could also contribute to these deaths. Yet, few studies have addressed these issues.

**Methods:**

We examined animal-bite reporting and assessed adherence to treatment guidelines at nine healthcare facilities in Haiti. We analyzed individual-level, de-identified patient data (demographic characteristics, geographic location, healthcare facility type, vaccine administration, and bite injury information) using descriptive analyses and logistic regression to examine factors associated with receiving PEP.

**Findings:**

During the 6 month study period, we found 2.5 times more animal-bite case-patients than reported by the national surveillance system (690 versus 274). Of the 690 animal-bite patients identified, 498 (72%) sought care at six PEP providing facilities. Of the case-patients that sought care, 110 (22%) received at least one rabies vaccine. Of the 110 patients, 60 (55%) received all five doses. Delays were observed for three events: when patients presented to a facility after an animal-bite (3.0 days, range: 0–34 days), when patients received their fourth dose (16.1 days, range: 13–52 days), and when patients received their fifth dose (29 days, range: 26–52). When comparing vaccination status and patient characteristics, we found a significant association for bite location (p < .001), severity rank score (p < .001), geographic location (p < .001), and healthcare facility type (p = .002) with vaccination.

**Conclusion:**

High levels of underreporting identified here are of concern since vaccine distribution may, in part, be based on the number of animal-bite cases reported. Given that the Haitian government provides PEP to the population for free and we found animal-bite victims are seeking care in a timely manner─ reducing rabies deaths is an achievable goal.

## Author summary

Human rabies deaths are preventable with timely provision of rabies post-exposure prophylaxis: wound treatment, rabies immunoglobulin, and vaccinations. In countries where resources are available, variability in healthcare seeking behaviors and lack of adherence to recommended treatment guidelines may also contribute rabies deaths. In our study, we examined animal-bite reporting to the national surveillance system, and patient’s adherence to treatment guidelines at nine healthcare facilities in Haiti, a high rabies burden country. Our study found 2.5 times more patients than reported by the national surveillance system. This underreporting can unintentionally impact rabies awareness and the allocation of resources to animal-bite victims. Furthermore, our study found that among patients who received care, a majority of them are seeking care in a timely manner, suggesting that reducing rabies deaths is an achievable goal in Haiti.

## Introduction

Approximately 59,000 people die from rabies annually worldwide [1]. Yet, these deaths are preventable with timely provision of rabies post-exposure prophylaxis (PEP): wound treatment, rabies immunoglobulin (RIG), and vaccinations [2]. Many of these deaths occur in canine rabies endemic countries where PEP may be limited or inaccessible to animal-bite victims [3]. In countries where PEP is available, variability in healthcare seeking behaviors and lack of adherence to recommended treatment guidelines could also contribute to these deaths. Research has shown that characteristics such as age, gender, and geographic location of residence are associated with seeking care for animal bites and receiving PEP [4–11]. Ensuring adequate and timely PEP administration for animal-bite patients in contact with a suspect rabid animal is one cornerstone of the World Health Organization’s (WHO) goal of eliminating rabies deaths. Therefore, understanding the management of rabies exposures is crucial to preventing future deaths.

Haiti is one of the last remaining countries in the Western Hemisphere with endemic canine rabies [1, 12–16]. From 2009–2012, an average of four canine and seven human rabies cases were reported by Haiti’s national surveillance system (NSS), however, studies have shown these reports underestimate the true burden of rabies [1, 13–15, 17]. One community-based surveillance program with active bite-case investigation, which was operated in three Haitian communes, detected more rabid animals than reported for the entire country by NSS [13]. Additionally, findings from an active case investigation estimated the rabies-associated mortality to be 0.67 cases/100,000 persons compared to 0.07cases/100,000 persons reported nationally [17]. Furthermore, a modeling study suggests 130 human deaths per year are attributable to rabies in Haiti [1]. Underreporting can unintentionally impact rabies awareness and the allocation of resources such as PEP to animal-bite victims [13, 17]. Few studies have examined treatment practices and adherence to vaccine schedules for animal-bite patients.

For this present study, we were interested in understanding animal-bite reporting, and adherence to treatment guidelines at healthcare facilities. To achieve this, we piloted active case-finding for all animal-bite patients presenting to selected health facilities in Haiti.

## Methods

### Healthcare facilities selection and PEP

We included nine healthcare facilities in our evaluation. These were selected by the Ministère de la Santé Publique et de la Population (MSPP). Three of these healthcare facilities were commune-level health centers and six facilities were hospitals. The facilities were located in six communes: Carrefour (2 sites; commune-level health center and hospital), Croix-des-Bouquets (1 site; hospital), Hinche (2 sites; commune-level health center and hospital), Leogane (1 site; hospital), Port-au-Prince (1 site; hospital), and Saint-Marc (2 sites; commune-level health center and hospital). All communes had at least one facility that provided PEP. Three communes (Carrefour, Hinche, Saint-Marc) also had facilities that did not provide PEP, but offered post-bite medical care. All facilities were located in high population density areas.

Rabies cell-cultured vaccines are donated to MSPP, often with assistance from PAHO, and are distributed to approximately 16 health facilities in Haiti (at least one facility per department). These vaccine are provided free of charge to patients when they are available. Stock-outs are frequent, in which case bite-victims must either purchase vaccines from private pharmacies at a cost of approximately $20 per dose, or travel to a different public facility to seek vaccine. Immunoglobulin is not provided at any public facilities, and are only available at private facilities for a fee or from the national stockpile with written permission from the Ministry of Health.

### Data, data reclassification, and dates

Our team visited each facility to conduct a retrospective review of all patients presenting for an animal-bite between April 1, 2015–September 30, 2015. We entered each patient’s individual level, de-identified data from the facilities’ paper-based animal-bite registries into an electronic database for analysis. Information from patients included demographic characteristics (age, gender), vaccine administration (dates of vaccination and dose number), and information related to the bite injury [bite location, bite type (e.g. multiple, single), animal, animal’s behavior]. WHO animal contact category was not collected at the facility, therefore it was unavailable for analysis. The data was reviewed and verified by a second individual for accuracy. Blank data were considered missing.

We defined patients receiving at least one of five intramuscular doses of the rabies vaccine as vaccinated. Patients receiving all five doses of vaccine were considered fully vaccinated. We categorized the nine facilities as PEP facilities (provided vaccine during the study period) or non-PEP facilities (provided wound treatment but no vaccine during the study period). None of the facilities provided rabies immunoglobulin during the study period. To categorize patient bite severity, we calculated a severity rank score for each patient as the sum of three variables: bite location (0 = missing, 1 = lower body, 2 = upper body, and 3 = multisite or head), bite type (0 = missing, 1 = single, and 2 = multiple), and animal’s behavior (0 = missing or calm, and 2 = aggressive). The severity rank score was then classified into: low (0 or 1 points), medium (2 or 3 points) and high (4 or greater points).

We calculated the number of days patients took to seek medical care and initiate vaccine. Deviations were defined by divergence from the recommended WHO PEP regimen and the degree of divergence was quantified by the number of off-schedule days. For example, a patient that waited to seek care three days after exposure would be considered a three-day deviation or a patient receiving their vaccine a day earlier than the recommended day would be considered a one-day deviation. Inaccurate dates such as documented date of vaccination occurring before the documented date of exposure were excluded from analysis.

### Statistical analysis

For our analysis, we were interested in factors that influenced rabies vaccination. To identify associations with vaccinated (at least one dose) and unvaccinated patients, we used a chi-square analysis and multivariable logistic regression. Our independent variable was patient vaccination status: yes or no. Dependent variables in the logistic regression models included: age, gender, animal type, severity rank score, facility location, and type of healthcare facility. To arrive at a final model, we used a backwards elimination. Due to small sample sizes among stratified characteristics we did not include interaction effects. Analyses were performed in R v3.3.2 statistical analysis software and the mapping was performed in ArcGIS v10.3.

### Surveillance data

To compare the findings from our site visits and case-finding in nine healthcare facilities, we compared the total number of animal-bite cases reported to Haiti’s NSS during the same study period (April 1, 2015–September 30, 2015) for the same communes.

### Ethical aspects

We obtained Institutional Review Board (IRB) approval from the Ministère de la Santé Publique et de la Population (MSPP) in Haiti and the IRB at the U.S. Centers for Disease Control and Prevention. The data were anonymous and de-identified.

## Results

### Location and patients captured at facility and national surveillance system

**Fig 1:**
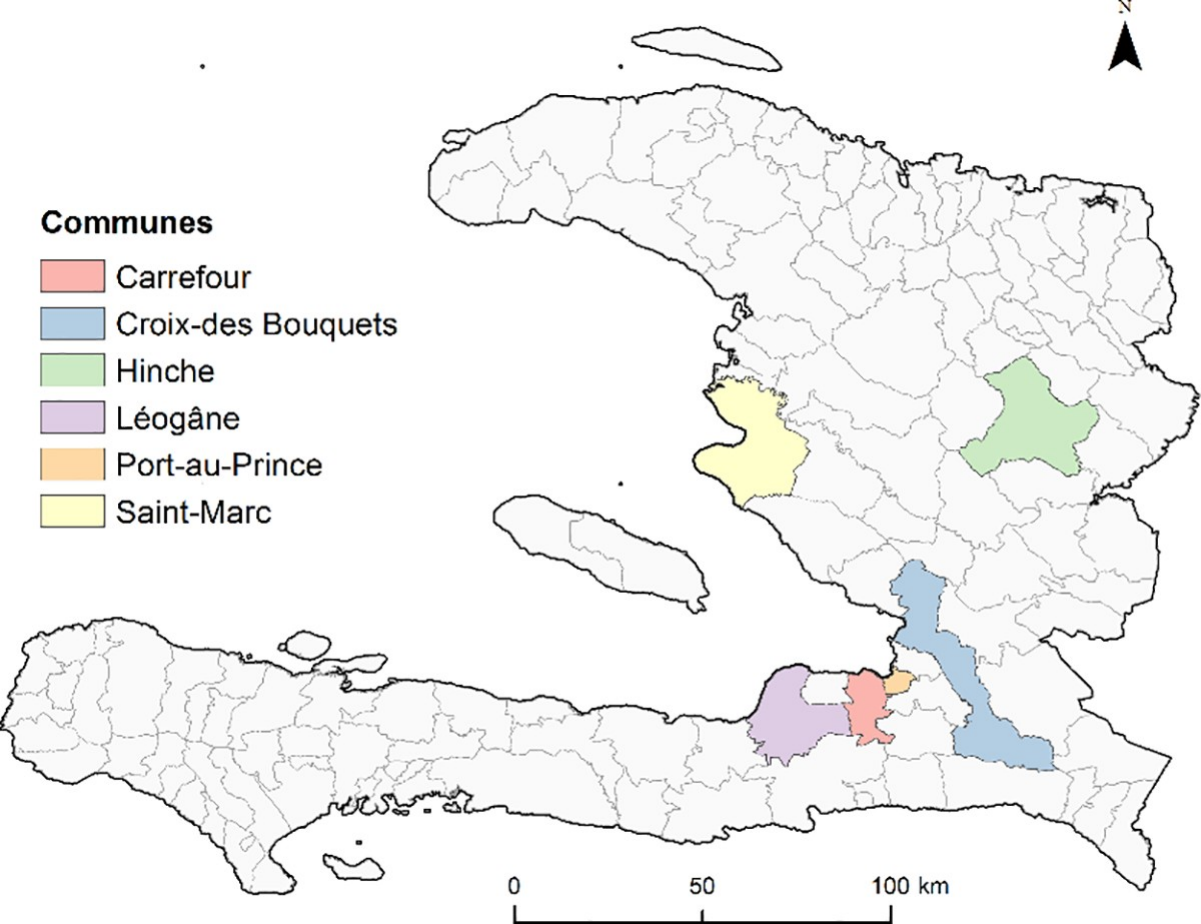
Location (A) and Number of Patients Captured by Healthcare Facility and National Surveillance System (B), Haiti 2015.

During the six-month period, our study identified 690 animal-bite patients at nine preselected healthcare facilities in six different communes (Fig 1, Table 1). The majority of animal-bite patients (60%) sought care in two of the six communes: Carrefour (n = 280, 40.6%) and Port-au-Prince (n = 134, 19.4%). Of the six communes where animal-bite patients presented for care, two communes, Saint-Marc and Leogane did not report any bite cases to the national surveillance system (NSS). In contrast with our findings, Haiti’s NSS reported only 274 animal-bite cases during the six-month period from these communes. Overall, 2.5 times more case-patients were identified from this study compared to the NSS (274 versus 690).

**Table 1:** Characteristics of patients presenting at nine healthcare facilities, Haiti 2015.

	Three Facilities Providing No PEP	Six Facilities Providing PEP		
	Vaccine not given, n (%)^+^ [%]^^^	Unvaccinated n, (%) [%]	Vaccinated n, (%) [%]	Chi-square
**No. Patients**	192 (27.8)	388 (56.2)	110 (15.9)	
**Age Group (years)**				χ^2^ = 4.98 p = 0.289
0–17	93 (32.0) [48.4]	148 (50.9) [38.1]	50 (17.2) [45.5]	
18–34	43 (23.2) [22.4]	114 (61.6) [29.4]	28 (15.1) [28.0]	
35–60	36 (22.8) [18.8]	98 (62.0) [25.3]	24 (15.2) [21.8]	
≥61	16 (36.4) [8.3]	20 (45.5) [5.2]	8 (18.2) [7.3]	
Missing	4 (33.3) [2.1]	8 (66.7) [2.1]	0	
**Gender**				χ^2^ = 3.56 p = 0.169
Female	94 (25.3) [47.4]	224 (60.4) [57.7]	53 (14.3) [48.2]	
Male	84 (27.6) [43.8]	163 (53.6) [42.0]	57 (18.8) [51.8]	
Missing	14 (93.3) [7.3]	1 (6.7) [0.3]	0	
**Bite Location**				χ^2^ = 34.0 p<0.001***
Multisite	11 (37.9) [5.7]	9 (31.0) [2.3]	9 (31.0) [8.2]	
Head	5 (71.4) [2.6]	1 (14.3) [2.6]	1 (14.3) [0.9]	
Upper Body	27 (24.5) [14.1]	51 (46.4) [13.1]	32 (29.1) [29.1]	
Lower Body	63 (20.6) [32.8]	191 (62.4) [49.2]	52 (17.0) [47.3]	
Missing	89 (36.1) [46.4]	136 (57.1) [35.1]	16 (6.72) [14.5]	
**Bite Type**				χ^2^ = 7.14 p = 0.028
Multiple	59 (25.5) [30.1]	127 (55.0) [32.7]	45 (19.5) [41.0]	
Single	36 (21.6) [18.8]	97 (58.1) [25.0]	34 (20.4) [30.9]	
Missing	97 (33.2) [50.5]	164 (56.1) [42.3]	31 (10.6) [28.2]	
**Animal**				χ^2^ = 7.94 p = 0.019
Dog	182 (28.5) [94.8]	338 (56.1) [87.1]	93 (15.4) [84.5]	
Other^a^	8 (29.6) [4.2]	10 (37.0) [2.6]	9 (33.3) [8.2]	
Missing	12 (20.0) [6.3]	40 (66.7) [10.3]	8 (13.3) [7.3]	
**Animal Behavior**				χ^2^ = 5.19 p = 0.075
Aggressive	96 (29.2) [50.0]	172 (52.3) [44.3]	61 (18.5) [55.5]	
Calm	1 (7.1) [0.5]	12 (85.7) [3.1]	1 (7.14) [0.9]	
Missing	95 (27.4) [49.5]	204 (58.8) [52.6]	48 (13.8) [43.6]	
**Severity Rank Score**^**b**^				χ^2^ = 20.38 p<0.001***
High	72 (26.0) [37.5]	142 (51.2) [36.6]	63 (22.7) [57.3]	
Medium	19 (10.2) [9.9]	133 (71.1) [34.3]	35 (18.7) [31.8]	
Low	101 (44.7) [52.6]	113 (50.0) [29.0]	12 (5.3) [10.9]	
**Location**				
Carrefour	137 (48.9) [71.4]	117 (41.8) [30.2]	26 (9.3) [23.7]	χ^2^ = 53.06 p<0.001***
Croix -des- Bouquet	No facility	48 (84.2) [12.4]	9 (15.8) [8.2]	
Hinche	16 (20.0) [8.3]	57 (71.3) [14.7]	7 (8.8) [6.4]	
Leogane	No facility	40 (83.3) [10.3]	8 (16.7) [7.3]	
Port-au-Prince	No facility	76 (56.7) [19.6]	58 (43.3) [52.7]	
Saint-Marc	39 (42.9) [20.3]	50 (54.9) [12.9]	2 (2.2) [1.8]	
**Healthcare Facility**				
Hospital	55 (12.1) [28.6]	298 (65.8) [76.9]	100 (22.1) [90.1]	χ^2^ = 9.76 p = 0.002**
Health clinic	137 (57.8) [71.4]	90 (38.0) [23.2]	10 (4.2) [9.9]	

Vaccinated individuals are those who have received at least 1 dose of rabies vaccine.

^+^Row percentages May not add up to 100% due to rounding.

^Column percentages may not add up to 100% due to rounding

^a^ The “Other” category includes cats human and pigs.

^b^ Severity Rank Score is based on the sum of points from the 3 variables: Bite Location, Bite Type and Animal Behavior. Severity Rank Score categories: Low = 0 or 1 points total points Medium = 2 or 3 total points High = 4 or more total points. Scores for Bite Location: 0 = Missing 1 = Lower Body 2 = Upper Body 3 = Multi or Head. Scores for Bite Type: 0 = Missing 1 = Single 2 = Multiple. Scores for Animal Behavior: 0 = Missing or Calm 2 = Aggressive.

### Vaccination completion

Of the 110 patients that were vaccinated, 60 (54.6%) patients received all five doses (Table 2). Vaccine completion increased with increasing age: 54% 0–17 years, 60.7% 18–34 years, 70.8% 35–60 years, and 87.5% 61 years and older.

**Table 2:** Vaccination completion by patient characteristics, Haiti 2015.

Characteristics	Vaccination				
	Dose 1	Dose 2	Dose 3	Dose 4	Dose 5
	No. Patients (%)	No. Patients (%)	No. Patients (%)	No. Patients (%)	No. Patients (%)
**Total**	110 (100)	79 (71.8)	73 (66.4)	70 (63.6)	60 (54.6)
**Gender**					
Female	53 (100)	38 (71.7)	34 (64.2)	33 (62.3)	30 (56.6)
Male	57 (100)	41 (71.9)	39 (68.4)	37 (64.9)	32 (56.1)
**Age group (years)**					
0–17	50 (100)	34 (68.0)	33 (66.0)	32 (64.0)	27 (54.0)
18–34	28 (100)	23 (82.1)	19 (67.9)	18 (64.3)	17 (60.7)
35–60	24 (100)	23 (95.8)	19 (79.2)	18 (75.0)	17 (70.8)
>60	8 (100)	7 (87.5)	7 (87.5)	7 (87.5)	7 (87.5)
**Severity rank score+**					
High	63 (100)	41 (65.1)	41 (65.1)	40 (63.5)	31 (49.2)
Medium	35 (100)	30 (85.7)	27 (77.1)	26 (74.3)	21 (60.0)
Low	12 (100)	8 (66.7)	5 (41.7)	4 (33.3)	2 (16.7)
**Location**					
Carrefour	26 (100)	19 (73.1)	19 (73.1)	19 (73.1)	19 (73.1)
Croix de Bouquet	9 (100)	9 (100)	9 (100)	8 (88.9)	8 (88.9)
Hinche	7 (100)	0	0	0	0
Leogane	8 (100)	5 (62.5)	4 (50.0)	3 (37.5)	0
Port-au-Prince	58 (100)	45 (77.6)	40 (69.0)	39 (67.2)	34 (58.6)
Saint-Marc	2 (100)	1 (50.0)	1 (50.0)	1 (50.0)	1 (50.0)

Patients living in Carrefour and Croix-des-Bouquets had vaccination completion rates over 70%, while Port-au-Prince and Saint-Marc were less than 60%. None of the 16 patients in Hinche and Leogane completed their fifth dose.

### Patient adherence and deviations to care

Patients sought care in a timely manner and generally followed the WHO PEP schedule (Table 3). Delays were observed for three events: when patients presented to a facility after an animal bite (3.0 days, range: 0–34 days), when patients received their fourth dose (16.1 days, range: 13–52 days), and when patients received their fifth dose (29 days, range: 26–52). No statistically significant delays were observed when data were stratified for characteristics such as age, gender, severity rank score, and area.

**Table 3:** Adherence to care among patients presenting with an animal-bite, Haiti 2015.

Difference between health events	WHO Recommendation	No. Patients^+^	Days
			Average, Range
Animal exposure and initial visit	Immediately	552	3.0, 0–34
Initial visit and vaccination of Dose 1	Day 0	122	0.9, 0–48
Dose 1 and vaccination of Dose 2	Day 3	71	3.5, 2–12
Dose 1 and vaccination of Dose 3	Day 7	64	7.6, 5–23
Dose 1 and vaccination of Dose 4	Day 14	62	16.1, 13–52
Dose 1 and vaccination of Dose 5	Day 28	54	29.0, 26–52

+ Number of patients with dates recorded.

When comparing deviations from the WHO recommendations, we noted that patients residing in Leogane were reported to have the largest deviation of an average of 6.3 days (range: 0–67). The mean deviations were not statistically different by age, gender, severity rank score, and location (Table 4).

**Table 4:** Deviations to care among patients presenting with an animal-bite, Haiti 2015.

Characteristics		No. Patients^+^	Days Average, Range	Chi-square
Age				p value = 0.89
	17 years and under	231	3.8, 0–17	
	18–35 years	156	3.7, 0–29	
	35–60 years	129	4.4, 0–37	
	61 years and older	33	4.8, 0–44	
Gender				p value = 0.82
	Female	309	4.0, 0–44	
	Male	239	4.2, 0–67	
Severity rank score^**+**^				p value = 0.39
	High	248	3.8, 0–34	
	Medium	192	3.7, 0–41	
	Low	119	4.9, 0–67	
Location				p value = 0.15
	Carrefour	231	3.1, 0–33	
	Croix de Bouquet	47	4.6, 0–29	
	Hinche	57	4.4, 0–44	
	Leogane	41	6.3, 0–67	
	Port au Prince	132	4.6, 0–34	
	St. Marc	51	3.5, 0–34	

^+^ Number of patients with available data.

### Patient characteristics at six facilities providing vaccine

Of the 690 animal-bite patients identified, 498 (72%) sought care at the six PEP facilities (Table 1). Of these, 110 (22%) animal-bite patients received at least one rabies vaccine. Rabies immunoglobulin was not documented for any of the patients.

When comparing vaccination status and variables of interest, we found a significant association (p < 0.05) for bite location (χ^2^ = 53.06; p < .001), severity rank score (χ^2^ = 20.38; p < .001), geographic location (χ^2^ = 53.06; p < .001), and healthcare facility type (χ^2^ = 9.76; p = .002). We found no association between vaccination status for age group (χ^2^ = 4.98; p = 0.289), gender (χ^2^ = 3.56; p = 0.169), bite type (χ^2^ = 7.14; p = 0.028), animal type (χ^2^ = 7.94; p = 0.019), and animal behavior (χ^2^ = 5.19; p = 0.075).

### Logistic regression

Results from our logistic regression analysis, comparing characteristics of vaccinated and unvaccinated patients at the six PEP facilities (n = 498) are shown in Table 5. The odds of receiving vaccine were 1.64 (95% CI: 1.01, 2.68) times greater for male patients than female patients. As the severity rank score increased (low versus high, medium versus low), so did the odds of receiving vaccine. The odds of receiving vaccine was 8.10 (95% CI: 3.26, 22.91) times greater for patients with a high severity compared to a low severity score (Table 5). The odds of receiving vaccine was 2.72 (95% CI: 1.04, 8.11) times greater for patients with a medium severity compared to a low severity score. Additionally, the odds of receiving vaccine were higher [OR: 5.14 (95% CI: 2.77, 9.85)] for patients residing in Port-au-Prince than Carrefour.

**Table 5:** Logistic regression analysis comparing characteristics of vaccinated and unvaccinated patients in six PEP providing facilities, Haiti, 2015.

	Univariate odds ratio (95% CI)	Adjusted odds ratio (95% CI)
**Age Group (years)** 0–17	Referent	-
18–34	0.75 (0.43, 1.29)	-
35–60	0.68 (0.37, 1.19)	-
>60	1.22 (0.47, 2.88)	-
**Gender**		
Female	Referent	Referent
Male	1.58 (1.02, 2.47)*	1.64 (1.01, 2.68)
**Animal**		
Dog	Referent	-
Other^a^	2.47 (0.88, 6.60)	-
**Severity Rank Score**^**b**^		
High	5.13 (2.47, 12.1)***	8.10 (3.26, 22.91)***
Medium	2.84 (1.30, 6.88)*	2.72 (1.04, 8.11)
Low	Referent	Referent
**Location**		
Carrefour	Referent	Referent
Croix de Bouquet	0.89 (0.36, 1.99)	2.41 (0.85, 6.65)
Hinche	0.69 (0.26, 1.63)	2.64 (0.82, 8.34)
Leogane	0.83 (0.23, 2.40)	1.24 (0.33, 3.88)
Port-au-Prince	3.18 (1.84, 5.57)***	5.14 (2.77, 9.85)***
Saint-Marc	0.18 (0.03, 0.63)*	0.27 (0.04, 0.99)
**Healthcare Facility**		
Hospital	Referent	-
Public Health	0.25 (0.10, 0.63)**	-

N = 447

Level of significance

*** p < 0.001

** p < 0.01

* p < 0.05

^a^ The “Other” category includes cats, human, and pigs.

^b^ Severity Rank Score is based on the sum of points from the 3 variables: Bite Location, Bite Type, and Animal Behavior

Severity Rank Score categories are: Low = 0 or 1 points total points, Medium = 2 or 3 total points, High = 4 or more total points.

## Discussion

We examined animal-bite treatment practices, patient adherence to vaccine, and animal-bite surveillance reporting in Haiti. We found patient adherence to the five-dose vaccine schedule was relatively high, but it was unclear why some high-risk patients went unvaccinated. Our active animal-bite case-finding uncovered issues with reporting compared to Haiti’s NSS; we identified 2.5 times more patients (690 patients from active case-finding versus 274 cases reported to NSS) with several communes not reporting to the NSS. Previous studies have also documented similar underreporting in Haiti [13, 17]. An active community investigation found two probable human rabies cases and 16 animal-bite victims that were not originally captured in NSS [17]. Underreporting is likely due to a lack of dedicated resources or a breakdown in reporting to NSS and limited healthcare seeking behavior among animal-bite victims. [13, 17]. Discrepancies in animal-bite cases may have severe consequences for vaccine allocation in areas that do not report or underreport cases.

In keeping with our findings, previous research found a higher frequency of animal bites among children [18]. Despite, the greater frequency of children found in our study, they had a lower initiation and completion rate compared to other age groups. In some instances, gender has also been documented as an important characteristic of healthcare-seeking behavior for animal bites and receiving vaccine [4]. Our study found a statistical difference between gender and vaccine administration but not vaccine adherence. Our experience working in Haiti leads us to believe that males are more likely to respond to an aggressive (potentially rabid) dog in a community, and may support why males in our study population are more likely to receive the first dose of vaccine.

Our logistic regression showed that independent predictors of receiving vaccination were gender, facility location, and severity rank score. While our logistic regression found higher odds of being vaccinated with a high severity rank score compared to a lower score, it is concerning that some high-risk patients did not initiate vaccination. For example, only half (n = 10) of patients with a multisite or head injury received vaccine. Given the lack of documentation, it is difficult to explain the reasons why this cohort did not receive vaccination. The dates suggest that there were no vaccine supply issues, as patients who were vaccinated presented at the same day or within a few days as those that did not receive vaccine. We believe a more comprehensive study is needed to better understand vaccine delivery and the rationale for not vaccinating high-risk individuals.

Adherence to the rabies vaccine schedule is associated with better survival after a rabies exposure. We found a surprisingly high adherence to completion of vaccine administration; 54% of animal-bite patients completed all five doses in our study. In contrast, lower adherence was reported in Iran (16.3%-18.7%), Tanzania (28%), and Cote d’Ivoire (47.3%) [6, 8, 19, 20]. Two studies in Nigeria had similar or higher completion coverage rates among animal-bite patients identified through a rabies lab registry and a pediatric hospital (60.7%) [21, 22]. However, we found a longer delay in seeking treatment after an exposure in our study population compared to similar studies in India, and Iran, where they reported a 24-hour delay of treatment after exposure among their study populations [5, 6, 10, 11]. Some studies found delays associated with age groups and gender, while our study did not [5, 6, 11]. The delays reported by those studies cited school-age patients not wanting to miss school in China, and women having less access to medical care due to cultural practices in Iran.

This study builds on the current rabies work in Haiti. Since the completion of this study, the Pan American Health Organization had developed and implemented a rabies training program for medical providers at health facilities. If this study was repeated at the same facilities, our findings could serve as a baseline on whether patient care, vaccine completion and adherence, and NSS reporting had improved. Three other studies found similar issues with adherence and compliance among bite-victims outside of Port-au-Prince [17, 23, 24]. Fenelon et al, reported that only 31% bite victims in Petionville (suburb outside of Port-au-Prince) initiated the vaccine series. Etheart et al, and Tran et al, found lack of compliance among bite-victims counselled by public health officials. Coupled with our findings, these studies suggests the need for comprehensive studies to understand the underlying factors for low compliance. This may help to identify strategies for increase vaccine uptake.

This study had limitations. First, the evaluation was based on a convenience sample, therefore may be potentially biased and not representative of the general population. For example, only one or two of the facilities in each commune were captured for this evaluation, therefore patients may have sought care elsewhere or were referred to another medical facility for vaccine. We attempted to address this by checking the names and birthdates for duplicate case-patients at each commune. We found one patient had sought care for treatment and vaccine at two different sites within the same commune. It is possible that other patients could have received vaccine or went home. Secondly, there was a large proportion of missing data for some of the variables. Similar studies have documented the same challenges with bite registries [7, 9].

In conclusion, we found important characteristics associated with vaccine administration and animal-bite exposures. The high levels of underreporting that we identified are of concern since the distribution of vaccine may in part based on the burden of animal-bite cases. Given that the Haitian government provides PEP to the population for free, we found animal-bite victims are generally seeking care in a timely manner. With improved surveillance and a better understanding of the underlying issues of low compliance ─ reducing rabies deaths is an achievable goal.
